# The Experiences of a Patient during Lithotomy before Anæsthesia

**Published:** 1905-05-27

**Authors:** 


### THE EXPERIENCES OF A PATIENT DURING LITHOTOMY BEFORE ANÆSTHESIA.

THE transactions of the Royal Medico-Chirurgical Society, now celebrating its centenary, include many papers of interest, and amongst them is one by Dr. Marcet which contains an account of the operation of lithotomy (performed in January 1812) given by the patient himself in the following words :—" My habit and constitution being good, it required little preparation of body and my mind was made up. When all parties had arrived I retired to my room for a minute and bent my knee in silent adoration and submission, and returning to the surgeons conducted them to the apartment in which the preparations had been made. The bandages, etc., being adjusted, I was prepared to receive a shock of pain of extreme violence, but so much had I over-rated it, that the first incisions did not even make me wince, although I had declared that it was not my intention to restrain such impulse, convinced that any effort of restraint could only lead to additional exhaustion. At subsequent moments, therefore, I did cry out, under the pain, but was allowed to have gone through the operation with great firmness. The forcing up of the staff prior to the introduction of the gorget gave me the first real pain, but this instantly subsided after the incision of the bladder was made, the rush of urine appearing to relieve it and soothe the wound. When the forceps was introduced the pain was again very considerable, and every movement of the instrument in endeavouring to find the stone increased it. Still, however, my mind was firm and confident, and although anxious, I was yet alive to what was going on. After several ineffectual attempts to grasp the stone, I heard the operator say in the lowest whisper, 'It is a little awkward, it lies under my hand, give me the curved forceps,' upon which he withdrew the others. Here, I think, I asked if there was anything wrong—or something to that purport—and was reanimated by the reply, conveyed in the kindest manner, 'Be patient, sir, it will soon be over.' When the other forceps was introduced, I had again to undergo the searching for the stone and heard Mr. Cline (surgeon to St. Thomas's Hospital, 1750–1827) say 'I have got it.' I had probably by this time conceived that the worst was over; but when the necessary force was applied to withdraw the stone the sensation was such as I cannot find words to describe. In addition to the positive pain there was something most peculiar in the feel. The bladder embraced the stone as firmly as the stone was itself grasped by the forceps : it seemed as if the whole organ was about to be torn out. The duration, however, of this really trying part of the operation was short, and when the words, 'Now, sir, it is all over,' struck my ear, the ejaculation of 'Thank God! thank God!' was uttered with a fervency and fulness of heart which can only be conceived. I am quite unable to describe my sensations at the moment. . . . There was a feeling of release, not from the pain of the operation, for that was gone and lost sight of, but from my enemy and tormentor, with a lightness and buoyancy of spirits elating my imagination to the belief that I was restored to perfect health as if by a miracle. I never heard what was the precise duration of this operation, but conceive it to have been between twelve and fifteen minutes. With respect to the pain I conceive that if it were possible to concentrate what I have often suffered in one night into the same space of time it would have been less endurable. Indeed this difference between the operation and one night's endurance of the stone was manifest. I have often been most distressingly reduced by the latter, but was not exhausted in the slightest degree by the former; at least my mind was firm throughout and my body was not sensibly enfeebled. Upon the whole should I be again similarly afflicted I should not hesitate in again submitting to the same mode of relief provided I could place myself in equally able hands."

It is pleasant to know that this plucky English gentleman speedily recovered. He ceased to keep his bed at the end of the second week; at the expiration of the third he was able to go downstairs and at the end of the month he walked near two miles. By the end of the sixth week he was able to ride on horseback and was in fact perfectly recovered.

